# Complications and survival after hybrid and fully minimally invasive oesophagectomy

**DOI:** 10.1093/bjsopen/zraa033

**Published:** 2021-02-11

**Authors:** M M K Veenstra, B M Smithers, E Visser, D Edholm, S Brosda, J M Thomas, D C Gotley, I G Thomson, B P L Wijnhoven, A P Barbour

**Affiliations:** Academy of Surgery, Faculty of Medicine, The University of Queensland, Brisbane, Queensland, Australia; Department of Surgery, Erasmus MC, University Medical Centre Rotterdam, Rotterdam, the Netherlands; Academy of Surgery, Faculty of Medicine, The University of Queensland, Brisbane, Queensland, Australia; Upper Gastrointestinal/Soft Tissue Unit, Princess Alexandra Hospital, Brisbane, Queensland, Australia; Mater Research Institute, Mater Health Services, Brisbane, Queensland, Australia; Upper Gastrointestinal/Soft Tissue Unit, Princess Alexandra Hospital, Brisbane, Queensland, Australia; Department of Surgery, University Medical Centre Utrecht, Utrecht, the Netherlands; Upper Gastrointestinal/Soft Tissue Unit, Princess Alexandra Hospital, Brisbane, Queensland, Australia; Department of Surgical Sciences, Uppsala University, Uppsala, Sweden; Diamantina Institute, Translational Research Institute, The University of Queensland, Queensland, Australia; Upper Gastrointestinal/Soft Tissue Unit, Princess Alexandra Hospital, Brisbane, Queensland, Australia; Mater Research Institute, Mater Health Services, Brisbane, Queensland, Australia; Academy of Surgery, Faculty of Medicine, The University of Queensland, Brisbane, Queensland, Australia; Upper Gastrointestinal/Soft Tissue Unit, Princess Alexandra Hospital, Brisbane, Queensland, Australia; Academy of Surgery, Faculty of Medicine, The University of Queensland, Brisbane, Queensland, Australia; Upper Gastrointestinal/Soft Tissue Unit, Princess Alexandra Hospital, Brisbane, Queensland, Australia; Department of Surgery, Erasmus MC, University Medical Centre Rotterdam, Rotterdam, the Netherlands; Academy of Surgery, Faculty of Medicine, The University of Queensland, Brisbane, Queensland, Australia; Upper Gastrointestinal/Soft Tissue Unit, Princess Alexandra Hospital, Brisbane, Queensland, Australia; Diamantina Institute, Translational Research Institute, The University of Queensland, Queensland, Australia

## Abstract

**Background:**

Minimally invasive oesophagectomy (MIO) is reported to produce fewer respiratory complications than open oesophagectomy. This study assessed differences in postoperative complications between MIO and hybrid MIO (HMIO) employing thoracoscopy and laparotomy, along with the influence of co-morbidities on postoperative outcomes.

**Methods:**

Patients with oesophageal cancer undergoing three-stage MIO or three-stage HMIO between 1999 and 2018 were identified from a prospectively developed database, which included patient demographics, co-morbidities, preoperative therapies, and cancer stage. The primary outcome was postoperative complications in the two groups. Secondary outcomes included duration of operation, blood transfusion requirement, duration of hospital stay, and overall survival.

**Results:**

There were 828 patients, of whom 722 had HMIO and 106 MIO, without significant baseline differences. Median duration of operation was longer for MIO (325 *versus* 289 min; *P* < 0.001), but with less blood loss (median 250 *versus* 300 ml; *P* < 0.001) and a shorter hospital stay (median 12 *versus* 13 days; *P* = 0.006). Respiratory complications were not associated with operative approach (31.1 *versus* 35.2 per cent for MIO and HMIO respectively; *P* = 0.426). Anastomotic leak rates (10.4 *versus* 10.2 per cent) and 90-day mortality (1.0 *versus* 1.7 per cent) did not differ. Cardiac co-morbidity was associated with more medical and surgical complications. Overall survival was associated with AJCC stage and co-morbidities, but not operative approach.

**Conclusion:**

MIO had a small benefit in terms of blood loss and hospital stay, but not in operating time. Oncological outcomes were similar in the two groups. Postoperative complications were associated with pre-existing cardiorespiratory co-morbidities rather than operative approach.

## Teaser

Some 828 patients who underwent either hybrid (HMIO) or fully minimally invasive oesophagectomy (MIO) were reviewed retrospectively for complications and survival. MIO had a small benefit in terms of blood loss and hospital stay, but not in operating time. Postoperative complications were associated with pre-existing cardiorespiratory co-morbidities rather than operative approach.

## Resumen

**Antecedentes:**

Se ha descrito que la esofaguectomía mínimamente invasiva (*minimally invasive oesophagectomy*, MIO) ocasiona menos complicaciones respiratorias que la esofaguectomía abierta. Este estudio evaluó diferencias en las complicaciones postoperatorias entre MIO y MIO híbrida (*hybrid* MIO, HMIO) en la que se utiliza toracoscopia y laparoscopia, así como la influencia de las comorbilidades en los resultados postoperatorios.

**Métodos:**

Se incluyeron en el estudio pacientes con cáncer de esófago, sometidos a MIO en 3 fases y/o HMIO en 3 fases, entre 1999 y 2018 e identificados a partir de una base de datos mantenida de forma prospectiva que incluía datos demográficos, comorbilidades, tratamientos preoperatorios y estadio del cáncer. El resultado primario era la presencia de complicaciones postoperatorias en los dos grupos. Los resultados secundarios incluyeron tiempo operatorio, necesidades transfusionales, estancia hospitalaria y supervivencia global.

**Resultados:**

Se seleccionaron 828 pacientes: 722 tratados mediante HMIO y 106 mediante MIO, sin diferencias basales significativas. La mediana del tiempo operatorio fue más larga en el grupo de MIO) (325 min *versus* 289 min, *P* < 0,001), mientras que la mediana de las pérdidas hemáticas (250 mL MIO *versus* 300 mL HMIO, *P* < 0,001) y mediana de estancia hospitalaria (12 días MIO, 13 días HMIO, *P* = 0,006) fue inferior. Las complicaciones respiratorias no se asociaron con el abordaje operatorio (35,2% HMIO, 31,1% MIO, *P* = 0,426). Las tasas de fuga anastomótica (10,2% HMIO, 10,4% MIO) o mortalidad a los 90 días (1,8% HMIO, 1% MIO) no difirieron. Las comorbilidades cardiacas se asociaron con un mayor número de complicaciones médicas y quirúrgicas. La supervivencia global se asoció con el estadio del AJCC y las comorbilidades, pero no con el abordaje operatorio.

**Conclusión:**

La práctica de MIO tiene un beneficio pequeño en las pérdidas hemáticas y estancia hospitalaria, pero no en el tiempo operatorio. Los resultados oncológicos fueron similares para cada grupo. Las complicaciones postoperatorias se asociaron con comorbilidades cardiorrespiratorias preexistentes más que con el abordaje operatorio.

## Introduction

Oesophageal cancer resection is complex surgery associated with significant postoperative morbidity and mortality rates^1^^,^^2^. Minimally invasive approaches have been introduced, aiming to reduce surgical trauma and postoperative morbidity^3–7^. These techniques include minimally invasive oesophagectomy (MIO)^6^^,^^8^, with chest and abdominal approaches performed by thoracoscopy and laparoscopy respectively; and hybrid MIO (HMIO), with either the thoracic or abdominal phase done by open surgery and the other component laparoscopically or thoracoscopically^9–11^. RCTs comparing open approaches with MIO^8^^,^^12^^,^^13^ and HMIO, involving laparoscopic gastric mobilization and an open thoracic approach^11^, have reported reduced blood loss, reduced overall morbidity, fewer respiratory complications, shorter hospital stay, and improved short-term quality of life after MIO or HMIO^11^^,^^12^^,^^14^^,^^15^. These trials have included anastomoses in the chest^13^ or neck^12^, and one of the MIO studies^12^ included robotic surgery.

Cohort studies, reviews and RCTs^15–19^ have not identified detrimental effects on resection margins, lymphadenectomy rates or long-term survival. MIO and HMIO have been associated with fewer respiratory complications, lower pain scores, and lower Clavien–Dindo grade III–IV complications than an open approach^8^^,^^11^^,^^17^^,^^18^^,^^20^, and have gained in popularity during the past decade^21^.

Co-morbidities, especially respiratory or cardiac pathologies, have been associated with postoperative complications and poor overall survival after oesophagectomy^22^^,^^23^. In addition, neoadjuvant chemotherapy^24^^,^^25^ or chemoradiotherapy^26^ are widely used but can increase the risk of complications such as anastomotic leak^27^. Minimally invasive approaches may overcome the detrimental impact of cardiorespiratory co-morbidities or neoadjuvant therapy, but studies addressing these interactions are lacking.

This study compared the impact of MIO and HMIO (thoracoscopy and open laparotomy) on operating time, blood loss, hospital complications, duration of hospital stay, and overall survival. The impact of cardiorespiratory co-morbidities was also assessed.

## Methods

Patients were identified from the prospectively created database of the Princess Alexandra Hospital Upper Gastro Intestinal Unit. HMIO has been used since 1993 and MIO since 1999, but more frequently since 2010. A trained research nurse reviewed patient history, graded complications, and entered information into the database. Patients were followed until death, or until 26 September 2019, when ethical approval was obtained (HREC/16/QPAH/614).

Patients were included in this study if they had either a three-phase (McKeown) MIO or HMIO (open abdomen) with cervical anastomosis. Patients were excluded if they underwent two-phase (Ivor Lewis) oesophagectomy or had undergone salvage surgery following definitive chemoradiotherapy. Patient demographics, preoperative co-morbidity, and preoperative treatment were recorded. Cardiac co-morbidity included arrhythmia, mild controlled congestive cardiac failure, controlled ischaemic heart disease, recent myocardial infarction, and cardiac failure. Respiratory co-morbidity included asthma, impaired respiratory tests, impaired exercise tolerance, and a forced expiratory volume (FEV1) < 1.5 litres. Intraoperative data and postoperative outcomes were evaluated, including complications, duration of hospital stay, and overall survival. These outcomes were then considered in the light of cardiac and respiratory co-morbidities.

### Surgery

Epidural catheters were placed routinely before operation for HMIO and optionally for MIO, and used for 4–5 days after surgery to manage pain. Full blood count, plasma electrolyte and liver function tests, ECG, lung function test, and CT were used routinely; CT–PET became standard practice from 2003. Endoscopic ultrasound imaging was done selectively. Patients with a gastro-oesophageal junctional tumour underwent staging laparoscopy.

Detailed descriptions of the HMIO and MIO procedures have already been published^9^^,^^11^^,^^28^^,^^29^. All anastomoses were performed through a left neck incision. Most procedures were undertaken in two hospitals by four surgeons who each performed both procedures. Feeding jejunostomy tubes were placed routinely in all patients. For MIO, the jejunostomy was placed via a 4-cm minilaparotomy extension of the supraumbilical port site. Early mobilization with physiotherapy, removal of nasogastric tubes, and reintroduction of diet followed the same protocol in both groups. Contrast swallows were used selectively.

### Complications

Postoperative complications were considered as surgical or medical. Individual complications were classified using the Clavien–Dindo system^30^ and standard reporting followed Esophagectomy Complications Consensus Group documentation^31^. Data entered before introduction of these classifications (2004 and 2015) were reclassified retrospectively. Surgical complications included bleeding, blood transfusion, vocal cord palsy, anastomotic leak, conduit necrosis, and wound infection. Postoperative bleeding was defined as blood loss requiring reoperation. Blood transfusion included infusions for blood loss during surgery or thereafter for postoperative bleeding. Vocal cord palsy was confirmed by laryngoscopy. An anastomotic leak was defined as any evidence of leakage clinically or on imaging. Wound infection was defined as erythema around a surgical wound or purulent discharge from a wound requiring antibiotic treatment and/or drainage. Medical complications included respiratory, cardiac arrhythmias/ischaemia, and bacteraemia/sepsis. Respiratory complications included atelectasis, pleural effusion, pneumonia, and acute respiratory distress syndrome; pneumonia was defined by a febrile illness with consistent clinical findings and radiological imaging. Complications were recorded during the hospital admission. Complications in a patient readmitted to hospital within 30 days of surgery were also recorded.

### Pathology and follow-up

Tumour were staged in accordance with the TNM staging system of the AJCC, seventh edition^32^. A resection margin was considered involved (R1), if there were tumour cells within 1 mm of a resection margin.

Patients were reviewed at 3-monthly intervals for 2 years, every 56 months for 3 years, and annually to 10 years. Assessment included history and examination with radiological and/or endoscopic assessment directed towards new symptoms or signs of recurrent disease. Information on those unable or unwilling to attend in person were obtained from the patient’s general practitioner or local hospital. Dates of death were obtained from family, hospital records, local medical officers or through death notices. Patients lost to follow-up were those who had surgery during or before 2018, who had not died from disease or after operation and who were disease-free at the time, but had less than 12 months of follow-up.

### Statistical analysis

Baseline and outcome scores were compared using the Mann–Whitney *U* test for continuous and discrete data with a non-normal distribution, and the χ^2^ test for categorical variables. *P* < 0.050 was deemed statistically significant in all analyses. Significant complications were adjusted for co-morbidities using linear regression analysis. Overall survival was calculated as the interval between surgery and date of death, or last follow-up, and was estimated using Kaplan–Meier and Cox regression methods. Disease-free survival was calculated as the interval between surgery and date of recurrence, or last follow-up, and was assessed using the same methods. The log rank (Mantel–Cox) test was used to assess log statistical significance and to identify factors associated with survival. Factors with *P* < 0.200 were subsequently included in multivariable analysis. Cox regression was used to build a multivariable survival model without correction for multiple testing. All data were analysed using SPSS^®^ version 25.0 for Windows^®^ (IBM, Armonk, New York, USA).

As HMIO was performed frequently between 2000 and 2010, with MIO gaining in popularity from 2010, a secondary propensity score matching analysis was performed to reduce cohort bias regarding this difference in time intervals. Matching was done using the R statistics MatchIt package version 3.0.2 (R Foundation for Statistical Computing, Vienna, Austria). The distance between patients was calculated using logistic regression, and the matched patient was found by the nearest-neighbour approach. Propensity score matching was based on the baseline variables age, sex, weight, history of smoking, co-morbidities, ASA fitness grade, histology, tumour location, AJCC stage, neoadjuvant treatment, fluorodeoxyglucose PET use, and year of surgery.

## Results

**Fig. 1:**
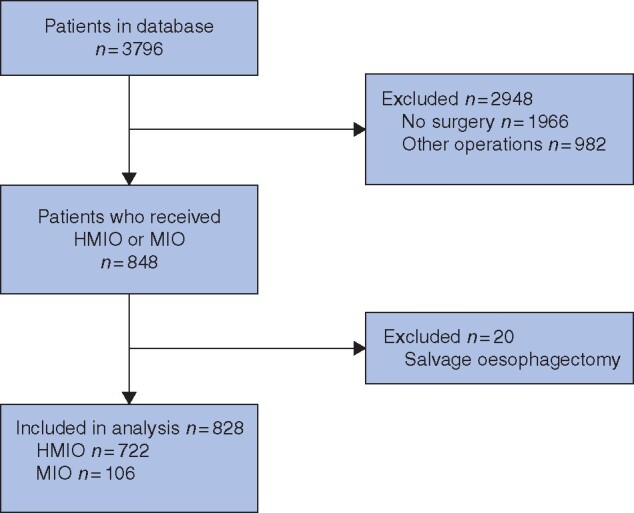
Study flow chart HMIO, hybrid minimally invasive oesophagectomy; MIO, minimally invasive oesophagectomy.

A total of 828 patients were included in this study, of whom 722 had HMIO and 106 MIO between August 1993 and September 2019 (*Fig. 1*). A total of 204 patients were propensity score-matched, 104 in each group. There were no differences in demographics, co-morbidity or preoperative treatment between the groups (*Table 1*).

**Table 1:** Baseline characteristics

	Total (*n *=* *828)	HMIO (*n *=* *722)	MIO (*n *=* *106)	*P* ^†^
**Age (years)** ^*^	64 (57–70)	64 (57–70)	64.5 (57–72)	0.456^‡^
**Sex ratio (M : F)**	681 : 147	595 : 127	86 : 20	0.748
**Cardiac co-morbidity**	286 (34.5)	241 (33.4)	45 (42.5)	0.067
**Respiratory co-morbidity**	201 (24.3)	177 (24.5)	24 (22.6)	0.674
**Smoking history**	510 (61.6)	450 (62.3)	60 (56.6)	0.258
**Tumour type**				0.617
Adenocarcinoma	632 (76.3)	548 (75.9)	84 (79.2)	
Squamous cell carcinoma	177 (21.4)	158 (21.9)	19 (17.9)	
Other	19 (2.3)	16 (2.2)	3 (2.8)	
**Tumour location**				0.110
Upper oesophagus	5 (0.6)	5 (0.7)	0 (0)	
Middle oesophagus	125 (15.1)	110 (15.3)	15 (14.2)	
Lower oesophagus	525 (63.6)	465 (64.6)	60 (56.6)	
Gastro-oesophageal junction	171 (20.7)	140 (19.4)	31 (29.2)	
**ASA fitness grade**				0.700
I–II	610 (73.9)	530 (73.7)	80 (75.5)	
III–IV	215 (26.1)	189 (26.3)	26 (24.5)	
**Clinical AJCC stage**				0.728
0	63 (7.6)	55 (7.6)	8 (7.5)	
I	131 (15.9)	115 (16.0)	16 (15.1)	
II	303 (36.7)	269 (37.4)	34 (32.1)	
III	327 (39.6)	279 (38.8)	48 (45.3)	
IV	2 (0.2)	2 (0.3)	0 (0)	
**Pathological AJCC stage**				0.447
0	37 (5.3)	29 (4.8)	8 (9)	
I	182 (26.3)	159 (26.2)	23 (26)	
II	202 (29.1)	181 (29.9)	21 (24)	
III	254 (36.7)	221 (36.5)	33 (38)	
IVa	18 (2.6)	16 (2.6)	2 (2)	
**Treatment**				0.471
Surgery alone	383 (46.3)	331 (45.8)	52 (49.1)	
Preoperative chemotherapy	160 (19.3)	137 (19.0)	23 (21.7)	
Preoperative CRT	285 (34.4)	254 (35.2)	31 (29.2)	

Values in parentheses are percentages unless indicated otherwise;

* values are median (i.q.r.). Data were incomplete for some variables. HMIO, hybrid minimally invasive oesophagectomy; MIO, minimally invasive oesophagectomy; CRT, chemoradiotherapy.

† χ^2^ test, except.

‡ Mann–Whitney *U* test.

### Complications

The overall medical and surgical complication rates were similar. There were no differences in respiratory complications or their severity, including rates of pneumonia (*Table 2*). There were more arrhythmias, typically atrial fibrillation, in the MIO group (32 of 106 (30.2 per cent) *versus* 130 of 722 (18.0 per cent); *P* = 0.003) but this was not significant after adjustment for preoperative cardiac co-morbidity (*P* = 0.099). Cardiac co-morbidities were also associated with other surgical and medical complications, including bleeding (9 of 286 (3.1 per cent) *versus* 5 of 542 (0.9 per cent) in patients with and without cardiac co-morbidity respectively; *P* = 0.018), sepsis (12 of 286 (4.2 per cent) *versus* 9 of 542 (1.7 per cent); *P* = 0.027), and conduit necrosis (8 of 286 (2.8 per cent) *versus* 5 of 542 (0.9 per cent); *P* = 0.039) (*Table S1*). Respiratory co-morbidity was associated with a higher 90-day mortality rate (5 of 201 (2.5 per cent) *versus* 5 of 627 (0.8 per cent) in patients with and without respiratory co-morbidity respectively; *P* = 0.048) (*Table S2*).

**Table 2:** Postoperative complications

	Total (*n *=* *828)	HMIO (*n *=* *722)	MIO (*n *=* *106)	**Univariable *P*** ^*^	Multivariable *P*^‡^
**Surgical**	205 (24.8)	183 (25.3)	22 (20.8)	0.306	
Bleeding	14 (1.7)	11 (1.5)	3 (2.8)	0.330	
Wound infection	65 (7.9)	58 (8.0)	7 (6.6)	0.609	
Vocal cord palsy	18 (2.2)	18 (2.5)	0 (0)	0.100	
Anastomotic leak	85 (10.3)	74 (10.2)	11 (10.4)	0.968	
Conduit necrosis	13 (1.6)	10 (1.4)	3 (2.8)	0.264	
**Medical**					
Respiratory	287 (34.7)	254 (35.2)	33 (31.1)	0.413	
Grade ≥ III	76 (9.2)	68 (9.4)	8 (7.5)	0.533	
Sepsis	21 (2.5)	16 (2.2)	5 (4.7)	0.126	
Cardiac	167 (20.2)	134 (18.6)	33 (31.1)	0.003	0.087
Arrhythmia	162 (19.6)	130 (18.0)	32 (30.2)	0.003	0.099
Ischaemia	10 (1.2)	9 (1.2)	1 (0.9)	0.790	
**Other**					
Reoperation	47 (5.7)	41 (5.7)	6 (5.7)	0.994	
In-hospital mortality	17 (2.1)	16 (2.2)	1 (1.0)	0.386	
30-day mortality	2 (0.2)	2 (0.2)	0 (0)	0.996^†^	
90-day mortality	–14 (1.7)	13 (1.8)	1 (1.0)	0.306^†^	

Values in parentheses are percentages. Data were incomplete for some variables. HMIO, hybrid minimally invasive oesophagectomy; MIO, minimally invasive oesophagectomy.

* χ^2^ test, except.

† logistic regression; model included all co-morbidities, medical complications, and ICU stay.

‡ Linear regression; significant complications were adjusted for all co-morbidities, medical complications, and ICU stay.

Because HMIO was performed over a long period compared with MIO, a secondary analysis was undertaken in which the cohort was propensity score-matched, leaving 104 patients in each group. Cardiac complications occurred more commonly in the MIO group, the majority being arrhythmia (31 of 104 (29.8 per cent) *versus* 14 of 104 (13.5 per cent); *P* = 0.017) (*Table S3*).

### Perioperative and pathological outcomes

Perioperative results are shown in *Table 3* and *Tables S1–S3*. MIO was associated with a longer abdominal (median 225 (i.q.r. 195–240) *versus* 185 (165–225) min; *P* < 0.001) and total (325 (278–360) *versus* 289 (240–330) min; *P* < 0.001) operating time, less blood loss during the abdominal phase (100 (50–200) *versus* 200 (100–300) ml; *P* < 0.001), reduced use of postoperative epidural pain relief (75.5 *versus* 97.3 per cent; *P* < 0.001), and a shorter hospital stay (median 12 (9–17) *versus* 13 (11–18) days; *P* = 0.006). There were no significant differences in R0 rate or number of lymph nodes examined between the two approaches.

**Table 3:** Perioperative results

	Total (*n *=* *828)	HMIO (*n *=* *722)	MIO (*n *=* *106)	**Univariable *P*** ^†^	**Multivariable *P*** ^§^
**Epidural**	760 (94.4)	680 (97.3)	80 (75.5)	< 0.001	< 0.001
**Duration of operation (min)** *					
Chest	90 (74–120)	90 (70–120)	90 (80–120)	0.129^‡^	
Abdomen	190 (167–235)	185 (165–225)	225 (195–240)	< 0.001^‡^	< 0.001
Total	296 (250–335)	289 (240–330)	325 (278–360)	< 0.001^‡^	< 0.001
**Blood loss (ml)** *					
Chest	100 (50–150)	100 (50–150)	100 (50–180)	0.418^‡^	
Abdomen	200 (100–300)	200 (100–300)	100 (50–200)	< 0.001^‡^	0.033
Total	300 (200–420)	300 (200–450)	250 (150–350)	< 0.001^‡^	0.179
**Blood transfusion**	132 (16.0)	121 (16.9)	11 (10.4)	0.090	
**Conversion**	21 (2.6)	18 (2.5)	3 (2.8)	0.865	
**Duration of hospital stay (days)** *	13 (11–17)	13 (11–18)	12 (9–17)	0.006^‡^	0.001
**Resection margin status**				0.809	
R0	716 (87.0)	623 (86.9)	93 (87.7)		
R1 or R2	107 (13.0)	94 (13.1)	13 (12.3)		
**No. of nodes removed** *					
Gastric	13 (10–18)	13 (9–18)	14 (11–18)	0.122^‡^	
Mediastinal	3 (1–5)	3 (1–5)	3 (1–5)	0.435^‡^	
Subcarinal	3 (1–5)	3 (1–5)	2 (1–6)	0.310^‡^	

Values in parentheses are percentages unless indicated otherwise;

* values are median (i.q.r.). Data were incomplete for some variables. HMIO, hybrid minimally invasive oesophagectomy; MIO, minimally invasive oesophagectomy.

† χ^2^ test, except.

‡ Mann–Whitney *U* test.

§ Linear regression; significant comparisons were adjusted for all co-morbidities, medical complications and ICU stay.

The relationships between co-morbidities and perioperative outcomes are summarized in *Tables S1* and *S2*. Co-morbidities were not associated with significant differences in operating times, blood loss, or duration of hospital stay. Patients with cardiac or respiratory co-morbidity were more likely to have R1–2 resection and fewer lymph nodes examined.

The propensity score-matched analysis showed that differences in median operating time (327 *versus* 300 min for MIO and HMIO respectively; *P* = 0.001) and hospital stay (12 and 14 days; *P* = 0.030) remained consistent (*Table S3*).

#### Survival and recurrence

**Fig. 2:**
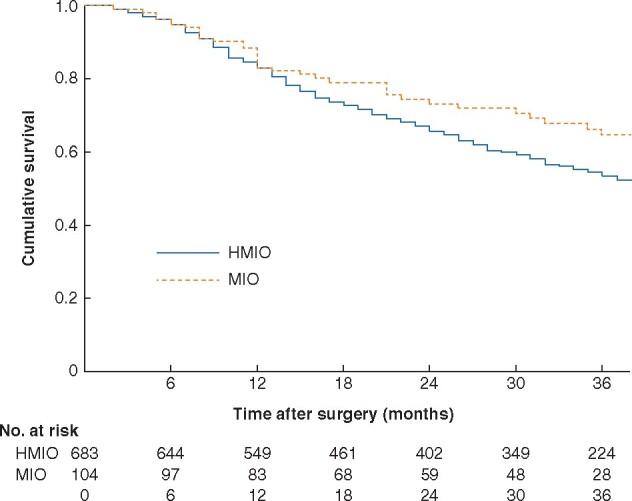
Comparison of survival after hybrid *versus* fully minimally invasive oesophagectomy HMIO, hybrid minimally invasive oesophagectomy; MIO, minimally invasive oesophagectomy. *P* = 0.262 (log rank test); *P* = 0.689 (multivariable Cox regression analysis adjusted for pathological AJCC stage, age, resection margin status, surgical approach, and respiratory and cardiac co-morbidity).

One patient was lost to follow-up owing to cancellation of the follow-up appointment. Median overall survival for the whole cohort was 48 (range 0–253) months, with 3- and 5-year overall survival rates of 55.4 and 47.3 per cent respectively. Median overall follow-up for survivors was 55 (range 0–253) months: 34 (2–205) months following MIO and 65 (0–253) months after HMIO. Median overall survival was 61 (2–205) months for MIO compared with 47 (0–253) months for HMIO (*P* = 0.262) (*Fig. 2*).

Univariable analyses showed pathological AJCC stage, age, R status, and respiratory and cardiac co-morbidity to be significant prognostic factors (*Table S4*). A multivariable survival analysis was undertaken including these univariable factors and operative approach. Age, AJCC stage, R status, and respiratory co-morbidity, but not operative approach, were independent prognostic factors for overall survival. No differences in recurrence patterns were found between the HMIO and MIO groups (*Tables S5–S7*). The 3-year disease-free survival rate was 63.4 per cent in the MIO group and 50.8 per cent in the HMIO group (*P* = 0.058). Three-year distant metastasis-free survival rates were 66.3 and 57.0 per cent respectively (*P* = 0.120), whereas 3-year locoregional recurrence-free survival rates were 82.7 and 79.0 per cent (*P* = 0.312). Multivariable analysis including the factors operative approach, AJCC stage, R status, and preoperative therapy showed that AJCC stage and R status, but not operative approach, were significantly associated with disease-free survival (*Tables S5–S7*).

## Discussion

The present study examined perioperative and survival outcomes after three-stage (McKeown) HMIO and MIO in a large, non-randomized consecutive patient cohort. The two operative approaches were associated with similar complications, and rates of these complications. Complications were, however, associated with pre-existing co-morbidities. Hospital stay was shorter for MIO, but no differences between the surgical groups were found in other perioperative outcomes, survival or recurrence.

In keeping with other studies^33–35^, the most common complications after oesophagectomy were respiratory, followed by surgical and cardiac complications. Two RCTs have reported fewer or less severe respiratory complications after MIO^8^, robot-assisted MIO^36^ or HMIO (with a laparoscopic abdominal phase)^11^ compared with open surgery, but comparisons of hybrid and total minimally invasive approaches have not been reported. The respiratory complication rates for MIO (31.1 per cent) and HMIO (35.2 per cent) were similar in the present series; the postoperative respiratory complications were driven more by respiratory co-morbidity, which is common in this population^23^^,^^37^^,^^38^. These data suggest that preoperative optimization of respiratory co-morbidity before MIO or HMIO is a more important consideration than the type of minimally invasive surgery.

Regarding other medical and surgical complications, including anastomotic leak, the surgical approaches were broadly equivalent. The postoperative morbidity rate was similar to that in other studies^11^^,^^39^^,^^40^ comparing either HMIO or MIO with an open procedure, and confirmed the safety of MIO and HMIO, in agreement with the TIME and MIRO trials^14^^,^^15^.

Cardiac arrhythmia was more frequent in the MIO group on univariable analysis, but this effect disappeared in multivariable analysis. No significant difference in the incidence of wound infection was found between the HMIO and MIO groups, probably reflecting the fact that the gastrointestinal tract is not opened during laparotomy along with prophylactic abdominal wound care. The median number of mediastinal lymph nodes dissected was similar in the two groups. Although the number of mediastinal nodes examined was low, nodal count is influenced by neoadjuvant therapy, the surgical procedure, and pathological specimen processing^41^^,^^42^.

Cardiac co-morbidities were associated with higher rates of postoperative complications including bleeding, sepsis, conduit necrosis, and cardiac complications, in line with a previous study^43^ that linked co-morbidities to a higher incidence of sepsis.

There were no significant differences in overall survival rates between the two operations. Respiratory co-morbidity was, however, an independent factor associated with poorer overall survival. These outcomes support the concept that both approaches are oncologically effective and safe to perform. The presence of cardiac or respiratory co-morbidities is an additional adverse prognostic factor for consideration in oesophageal cancer management^22^^,^^23^.

Although based on a large cohort of patients with use of a specifically created database, there are limitations to this analysis. It was an observational, non-randomized study, performed in a single institution with a long period of data acquisition. Patients who underwent MIO accounted for only 12.8 per cent of procedures. To minimize selection bias, a propensity score matching analysis was performed, but low numbers may have limited its statistical power to identify differences. However, the data suggest that other differences are not large and may not be clinically relevant. There was no correction for multiple testing, so some differences could still have been down to chance. The data are representative of a population in which adenocarcinoma dominates, so may not be applicable in countries where squamous cell cancer is more common.

This study has confirmed the safety and feasibility of MIO, with short- and long-term oncological outcomes comparable to those of HMIO. There was no major impact from the introduction of laparoscopic gastric mobilization. Cardiorespiratory co-morbidities have a far greater impact on postoperative outcomes than the surgical approach.

## Funding

Swedish Society of Medicine

## Supplementary Material

zraa033_Supplementary_Data
