# Supplementary material for: Complications and survival after hybrid and fully minimally invasive oesophagectomy
Source: BJS Open. 2021 Feb 11;5(1):zraa033. doi: 10.1093/bjsopen/zraa033 (PMC7893474; doi:10.1093/bjsopen/zraa033)
Supplement: zraa033_Supplementary_Data [file zraa033_supplementary_data.docx]

**Table S1 Cardiac co-morbidity**

| Factor | Total  (*n* = 828) | No cardiac comorbidity (*n* = 542) | Cardiac comorbidity (*n* = 286) | *p* Value^a^ |
| --- | --- | --- | --- | --- |
| Epidural | 760 (94.4%) | 496 (93.6%) | 264 (96.0%) | 0.157 |
| Primary outcome |  |  |  |  |
| Postoperative complications |  |  |  |  |
| Surgical | 205 (24.8%) | 134 (24.7%) | 71 (24.8%) | 0.974 |
| Bleeding | 14 (1.7%) | 5 (0.9%) | 9 (3.1%) | **0.018** |
| Wound infection | 65 (7.9%) | 42 (7.7%) | 23 (8.0%) | 0.882 |
| Vocal cord palsy | 18 (2.2%) | 11 (2.0%) | 7 (2.4%) | 0.695 |
| Anastomotic leak | 85 (10.3%) | 55 (10.1%) | 30 (10.5%) | 0.878 |
| Conduit necrosis | 13 (1.6%) | 5 (0.9%) | 8 (2.8%) | **0.039** |
| Reoperation | 47 (5.7%) | 29 (5.4%) | 18 (6.3%) | 0.577 |
| Medical |  |  |  |  |
| Respiratory complications | 287 (34.7%) | 181 (33.4%) | 106 (37.1%) | 0.292 |
| Grade ≥ III | 76 (9.2%) | 40 (7.4%) | 36 (12.6%) | **0.014** |
| Sepsis | 21 (2.5%) | 9 (1.7%) | 12 (4.2%) | **0.027** |
| Cardiac | 167 (20.2%) | 88 (16.2%) | 79 (27.6%) | **0.000** |
| Arrhythmia | 162 (19.6%) | 86 (15.9%) | 76 (26.6%) | **0.000** |
| Ischemia | 10 (1.2%) | 4 (0.7%) | 6 (2.1%) | 0.088 |
| Secondary outcomes |  |  |  |  |
| Intraoperative data |  |  |  |  |
| OR time, minutes, median (IQR) |  |  |  |  |
| Chest | 90.0 (74-120) | 90.0 (70-120) | 90.0 (75-120) | 0.052^b^ |
| Abdomen | 190.0 (167-235) | 190.0 (165-230) | 200.0 (180-240) | **0.010^b^** |
| Total | 296.0 (250-335) | 285.0 (240-330) | 300.0 (265-340) | **0.007^b^** |
| Blood loss, mL, median (IQR) |  |  |  |  |
| Chest | 100.0 (50-150) | 100.0 (50-150) | 100.0 (50-150) | **0.038^b^** |
| Abdomen | 200.0 (100-300) | 200.0 (100-300) | 200.0 (100-300) | 0.191^b^ |
| Total | 300.0 (200-420) | 300.0 (200-400) | 300.0 (200-450) | 0.086^b^ |
| Blood transfusion | 132 (16.0%) | 74 (13.7%) | 58 (20.4%) | **0.012** |
| Conversion | 21 (2.6%) | 8 (1.5%) | 13 (4.7%) | **0.007** |
| Postoperative data |  |  |  |  |
| Hospital days, median (IQR) | 13.0 (11-17) | 13.0 (11-17) | 14.0 (11-18) | 0.301^b^ |
| R status |  |  |  | 0.792 |
| 0 | 716 (87.0%) | 471 (87.2%) | 245 (86.6%) |  |
| 1 or 2 | 107 (13.0%) | 69 (12.8%) | 38 (13.4%) |  |
| Nodes removed, median (IQR) |  |  |  |  |
| Gastric | 13.0 (10-18) | 14.0 (10-19) | 13.0 (9-17) | **0.044^b^** |
| Mediastinal | 3.0 (1-5) | 3.0 (1-5) | 3.0 (1-5) | 0.359^b^ |
| Subcarinal | 3.0 (1-5) | 3.0 (1-5) | 3.0 (0-5) | 0.312^b^ |
| In hospital mortality | 17 (2.1%) | 6 (1.1%) | 11 (3.9%) | **0.008** |
| 30-day mortality | 2 (0.2%) | 0 (0.0%) | 2 (0.7%) | 0.993^c^ |
| 90-day mortality | 14 (1.7%) | 6 (1.1%) | 8 (2.8%) | 0.551^c^ |
| Survival data | **Total**  **(*n* = 816)** | **No cardiac comorbidity**  **(*n* = 536)** | **Cardiac comorbidity**  **(*n* = 282)** |  |
| Overall survival, months, median (SE) | 51.0 (5.8) | 56.0 (5.1) | 34.0 (4.9) | **0.008^d^** |

1. Chi-square test
2. Mann-Whitney U test
3. Logistic regression. Model included all comorbidities, medical complications and ICU stay.
4. Log-Rank (Mantel Cox)

**Table S2 Respiratory co-morbidity**

| Factor | Total  (*n* = 828 ) | No respiratory comorbidity (*n* = 627) | Respiratory comorbidity (*n* = 201) | *p* Value^a^ |
| --- | --- | --- | --- | --- |
| Epidural | 760 (94.4%) | 581 (95.1%) | 179 (92.3%) | 0.136 |
| Primary outcome |  |  |  |  |
| Postoperative complications |  |  |  |  |
| Surgical | 205 (24.8%) | 158 (25.2%) | 47 (23.4%) | 0.604 |
| Bleeding | 14 (1.7%) | 10 (1.6%) | 4 (2.0%) | 0.705 |
| Wound infection | 65 (7.9%) | 49 (7.8%) | 16 (8.0%) | 0.947 |
| Vocal cord palsy | 18 (2.2%) | 16 (2.6%) | 2 (1.0%) | 0.188 |
| Anastomotic leak | 85 (10.3%) | 63 (10.0%) | 22 (10.9%) | 0.715 |
| Conduit necrosis | 13 (1.6%) | 10 (1.6%) | 3 (1.5%) | 0.919 |
| Reoperation | 47 (5.7%) | 41 (6.5%) | 6 (3.0%) | 0.058 |
| Medical |  |  |  |  |
| Respiratory complications | 287 (34.7%) | 208 (33.2%) | 79 (39.3%) | 0.112 |
| Grade ≥ III | 76 (9.2%) | 52 (8.3%) | 24 (11.9%) | 0.119 |
| Sepsis | 21 (2.5%) | 15 (2.4%) | 6 (3.0%) | 0.642 |
| Cardiac | 167 (20.2%) | 120 (19.1%) | 47 (23.4%) | 0.192 |
| Arrhythmia | 162 (19.6%) | 116 (18.5%) | 46 (22.9%) | 0.173 |
| Ischemia | 10 (1.2%) | 7 (1.1%) | 3 (1.5%) | 0.671 |
| Secondary outcomes |  |  |  |  |
| Intraoperative data |  |  |  |  |
| OR time, minutes, median (IQR) |  |  |  |  |
| Chest | 90.0 (74-120) | 90.0 (70-120) | 90.0 (78-120) | **0.001^b^** |
| Abdomen | 190.0 (167-235) | 190.0 (165-230) | 207.0 (173-240) | 0.077^b^ |
| Total | 296.0 (250-335) | 285.0 (245-330) | 300.0 (265-350) | **0.002^b^** |
| Blood loss, mL, median (IQR) |  |  |  |  |
| Chest | 100.0 (50-150) | 100.0 (50-150) | 100.0 (50-180) | **0.010^b^** |
| Abdomen | 200.0 (100-300) | 200.0 (100-300) | 195.0 (100-300) | 0.390^b^ |
| Total | 300.0 (200-420) | 300.0 (200-420) | 300.0 (200-425) | 0.093^b^ |
| Blood transfusion | 132 (16.0%) | 88 (14.1%) | 44 (21.9%) | **0.009** |
| Conversion | 21 (2.6%) | 13 (2.1%) | 8 (4.1%) | 0.130 |
| Postoperative data |  |  |  |  |
| Hospital days, median (IQR) | 13.0 (11-17) | 13.0 (11-18) | 13.0 (10-17) | 0.979^b^ |
| R status |  |  |  | **0.032** |
| 0 | 716 (87.0%) | 550 (88.4%) | 166 (82.6%) |  |
| 1 or 2 | 107 (13.0%) | 72 (11.6%) | 35 (17.4%) |  |
| Nodes removed, median (IQR) |  |  |  |  |
| Gastric | 13.0 (10-18) | 14.0 (10-19) | 12.0 (8-17) | **0.004^b^** |
| Mediastinal | 3.0 (1-5) | 3.0 (1-5) | 3.0 (1-6) | 0.188^b^ |
| Subcarinal | 3.0 (1-5) | 3.0 (1-5) | 2.0 (0-4) | **0.003^b^** |
| In hospital mortality | 17 (2.1%) | 10 (1.6%) | 7 (3.5%) | 0.099 |
| 30-day mortality | 2 (0.2%) | 0 (0.0%) | 2 (1.0%) | 0.995^c^ |
| 90-day mortality | 14 (1.7%) | 5 (0.8%) | 9 (4.5%) | **0.048^c^** |
| Survival data | **Total**  **(*n* = 818)** | **No respiratory comorbidity (*n* = 620)** | **Respiratory comorbidity (*n* = 198)** |  |
| Overall survival, months, median (SE) | 51.0 (5.8) | 61.0 (5.3) | 32.0 (6.1) | **0.002^d^** |

1. Chi-square test
2. Mann-Whitney U test
3. Logistic regression. Model included all comorbidities, medical complications and ICU stay.
4. Log Rank (Mantel-Cox)

**Table S3 Propensity score-matched cohort results**

| Factor | Total  (*n* = 208) | HMIE group (*n* = 104) | MIE group (*n* = 104) | Univariable *p* Value^a^ | Multivariable *p* Value^e^ |
| --- | --- | --- | --- | --- | --- |
| Primary outcome |  |  |  |  |  |
| Postoperative complications |  |  |  |  |  |
| Surgical | 51 (24.5%) | 29 (27.9%) | 22 (21.2%) | 0.259 |  |
| Bleeding | 4 (1.9%) | 1 (1.0%) | 3 (2.9%) | 0.313 |  |
| Wound infection | 12 (5.8%) | 4 (4.8%) | 7 (6.7%) | 0.552 |  |
| Vocal cord palsy | 5 (2.4%) | 5 (4.8%) | 0 (0.0%) | **0.024** | **0.021** |
| Anastomotic leak | 29 (13.9%) | 18 (17.3%) | 11 (10.6%) | 0.161 |  |
| Conduit necrosis | 3 (1.4%) | 0 (0.0%) | 3 (2.9%) | 0.081 |  |
| Reoperation | 12 (5.8%) | 6 (5.8%) | 6 (5.8%) | 1.000 |  |
| Medical |  |  |  |  |  |
| Respiratory complications | 70 (33.7%) | 37 (35.6%) | 33 (31.7%) | 0.557 |  |
| Grade ≥ III | 17 (8.2%) | 9 (8.7%) | 8 (7.7%) | 0.800 |  |
| Sepsis | 7 (3.4%) | 2 (1.9%) | 5 (4.8%) | 0.249 |  |
| Cardiac | 45 (21.6%) | 14 (13.5%) | 31 (29.8%) | **0.004** | **0.017** |
| Arrhythmia | 44 (21.2%) | 14 (13.5%) | 30 (28.8%) | **0.007** | **0.027** |
| Ischemia | 1 (0.5%) | 0 (0.0%) | 1 (0.5%) | 0.316 |  |
| Secondary outcomes |  |  |  |  |  |
| Intraoperative data |  |  |  |  |  |
| Epidural | 175 (84.5%) | 96 (93.2%) | 79 (76.0%) | **0.001** | **0.000** |
| OR time, minutes, median (IQR) |  |  |  |  |  |
| Chest | 90.0 (78-120) | 90.0 (70-120) | 90.0 (80-120) | 0.195^b^ |  |
| Abdomen | 210.0 (180-240) | 185.0 (165-240) | 225.0 (195-240) | **0.000^b^** | **0.000** |
| Total | 310.0 (270-360) | 300.0 (250-330) | 327.5 (280-360) | **0.001^b^** | **0.001** |
| Blood loss, mL, median (IQR) |  |  |  |  |  |
| Chest | 100.0 (50-150) | 80.0 (50-120) | 100.0 (50-180) | **0.020^b^** | **0.001** |
| Abdomen | 150.0 (78-245) | 190.0 (100-300) | 100.0 (50-200) | **0.000^b^** | 0.313 |
| Total | 250.0 (150-400) | 250.0 (150-400) | 245.0 (150-350) | 0.180^b^ |  |
| Blood transfusion | 20 (9.6%) | 10 (9.6%) | 10 (9.6%) | 1.000 |  |
| Conversion | 3 (1.4%) | 0 (0.0%) | 3 (2.9%) | 0.081 |  |
| Postoperative data |  |  |  |  |  |
| Hospital days, median (IQR) | 13.0 (10-18) | 14.0 (11-18) | 12.0 (9-17) | 0.051^b^ | **0.030** |
| R status |  |  |  | 0.529 |  |
| 0 | 178 (86.0%) | 87 (84.5%) | 91 (87.5%) |  |  |
| 1 or 2 | 29 (14.0%) | 16 (15.5%) | 13 (12.5%) |  |  |
| Nodes removed, median (IQR) |  |  |  |  |  |
| Gastric | 15.0 (11-20) | 15.0 (12-21) | 14.0 (11-18) | 0.058^b^ |  |
| Mediastinal | 3.0 (1-5) | 3.0 (1-5) | 3.0 (1-5) | 0.990^b^ |  |
| Subcarinal | 2.0 (1-5) | 3.0 (1-5) | 2.0 (1-6) | 0.524^b^ |  |
| In hospital mortality | 2 (1.0%) | 1 (1.0%) | 1 (1.0%) | 1.000 |  |
| 30-day mortality | 0 (0.0%) | 0 (0.0%) | 0 (0.0%) | 1.000^c^ |  |
| 90-day mortality | 6 (2.9%) | 2 (1.9%) | 4 (3.8%) | 0.427^c^ |  |
| Survival data |  |  |  |  |  |
| Overall survival, months, median (SE) | 74.0 (9.7) | 83.0 (11.0) | 63.0 (10.0) | 0.444^d^ |  |

1. Chi-square test
2. Mann-Whitney U test
3. Logistic regression. Model included all comorbidities, medical complications and ICU stay.
4. Log-Rank (Mantel Cox)
5. Linear regression. Significant complications were adjusted for all comorbidities, medical complications and ICU stay.

**Table S4 Prognostic factors for overall survival**

| **Factor** | **Median OS (months)** | **Univariable p-value^a^** | **Multivariable p-value^b^** | **Hazard ratio** | **95% CI** |
| --- | --- | --- | --- | --- | --- |
| Age | - | **0.002** | **0.005** | **1.02** | **1.01-1.03** |
| AJCC stage |  | **0.000** | **0.000** |  |  |
| 0 (n = 37) | 163 |  |  | **1.0** |  |
| I (n = 182) | 158 |  |  | **4.13** | **1.01-16.91** |
| II (n = 202) | 47 |  |  | **9.75** | **2.41-39.46** |
| III (n = 254) | 21 |  |  | **19.91** | **4.93-80.35** |
| Iva (n = 18) | 8 |  |  | **76.94** | **17.30-342.28** |
| Cardiac comorbidity |  | 0.008 | 0.051 |  |  |
| No (n = 542) | 56 |  |  |  |  |
| Yes (n = 286) | 34 |  |  |  |  |
| Operative approach |  | 0.083 | 0.308 |  |  |
| HMIE (n = 722) | 47 |  |  |  |  |
| MIE (n = 106) | 63 |  |  |  |  |
| R status |  | **0.000** | **0.003** |  |  |
| R0 (n = 716) | 68 |  |  |  |  |
| R1 or R2 (n = 107) | 14 |  |  | **1.53** | **1.15-2.03** |
| Respiratory comorbidity |  | **0.001** | **0.01** |  |  |
| No (n = 627) | 56 |  |  |  |  |
| Yes (n = 201) | 32 |  |  | **1.34** | **1.07-1.67** |
| Diabetes |  | 0.748 | 0.698 | 1.05 |  |
| No (n =742) | 49 |  |  |  |  |
| Yes (n = 86) | 45 |  |  |  |  |
| Treatment |  | 0.065 | 0.437 |  |  |
| Surgery (n = 383) | 54 |  |  |  |  |
| Preop chemo (n = 160) | 50 |  |  |  |  |
| Preop CRT (n = 285) | 42 |  |  |  |  |

1. Log Rank (Mantel-Cox)
2. Cox regression (all factors above are covariates)

**Table S5 Disease-free survival**

| **Clinicopathological Variable** | **Univariable Factors** | | **Multivariable Factors** | | |
| --- | --- | --- | --- | --- | --- |
|  | **3-year survival (%)** | ***P* Value^a^** | **Hazard Ratio** | **95% CI** | ***P* Value^b^** |
| Surgery HMIE (n = 671)  MIE (n = 106) | 50.8  63.4 | 0.058 | 0.775 | 0.544 – 1.103 | 0.157 |
| **AJCC stage** 0 (n = 36)  I (n = 166)  II (n = 198)  III (n = 243)  IV (n = 18) | 88.3  83.1  50.3  19.7  0.0 | **0.000** | -  1.915  6.168  14.452  169.099 | -  0.584 – 6.275  1.952 – 19.491  4.584 – 45.563  48.485–590.087 | -  0.284  **0.002**  **0.000**  **0.000** |
| **R status** R0 (n = 678)  R1/2 (n = 96) | 57.7  15.4 | **0.000** | 1.586 | 1.194 – 2.107 | **0.001** |
| **Preoperative therapy**  No (n = 349)  Chemo (n = 156)  CRT (n = 272) | 56.9  50.1  47.3 | **0.002** | -  0.795  1.140 | -  0.601 – 1.052  0.891 – 1.459 | -  0.108  0.297 |

1. Log Rank (Mantel-Cox)
2. Cox regression

Multivariable model included AJCC stage, age, R status, preoperative therapy, respiratory co-morbidity, cardiac co-morbidity and surgical approach.

**Table S6 Distant metastasis-free survival**

| **Clinicopathological Variable** | **Univariable Factors** | | **Multivariable Factors** | | |
| --- | --- | --- | --- | --- | --- |
|  | **3-year survival (%)** | ***P* Value^a^** | **Hazard Ratio** | **95% CI** | ***P* Value^b^** |
| Surgery HMIE (n = 672)  MIE (n = 106) | 57.0  66.3 | 0.120 | 0.807 | 0.551 – 1.182 | 0.271 |
| **AJCC stage** 0 (n = 36)  I (n = 166)  II (n = 198)  III (n = 243)  IV (n = 18) | 93.0  85.0  57.9  25.6  0.1 | **0.000** | -  2.375  7.333  17.766  217.578 | -  0.563 – 10.018  1.799 – 29.894  4.371 – 72.206  48.699-972.089 | -  0.239  **0.005**  **0.000**  **0.000** |
| **R status** R0 (n = 679)  R1/2 (n = 96) | 63.0  20.6 | **0.000** | 1.526 | 1.118 – 2.083 | **0.008** |
| **Preoperative therapy**  No (n = 350)  Chemo (n = 156)  CRT (n = 272) | 61.4  56.3  55.3 | **0.036** | -  0.717  1.041 | -  0.526 – 0.976  0.794 – 1.363 | -  **0.035**  0.773 |

1. Log Rank (Mantel-Cox)
2. Cox regression

Multivariable model included AJCC stage, age, R status, preoperative therapy, respiratory co-morbidity, cardiac co-morbidity and surgical approach.

**Table S7 Locoregional recurrence-free survival**

| **Clinicopathological Variable** | **Univariable Factors** | | **Multivariable Factors** | | |
| --- | --- | --- | --- | --- | --- |
|  | **3-year survival (%)** | ***P* Value^a^** | **Hazard Ratio** | **95% CI** | ***P* Value^b^** |
| Surgery HMIE (n = 672)  MIE (n = 106) | 79.0  82.7 | 0.312 | 0.786 | 0.439 – 1.407 | 0.417 |
| **AJCC stage** 0 (n = 36)  I (n = 166)  II (n = 198)  III (n = 243)  IV (n = 18) | 100.0  93.2  79.4  52.5  0.0 | **0.000** | -  N/A  N/A  N/A  N/A | -  N/A  N/A  N/A  N/A | -  N/A  N/A  N/A  N/A |
| **R status** R0 (n = 679)  R1/2 (n = 96) | 83.1  45.9 | **0.000** | 1.880 | 1.172 – 3.016 | **0.009** |
| **Preoperative therapy**  No (n = 350)  Chemo (n = 156)  CRT (n = 272) | 84.6  75.9  74.4 | **0.002** | -  1.177  1.727 | -  0.731 – 1.895  1.122 – 2.658 | -  0.502  **0.013** |

1. Log Rank (Mantel-Cox)
2. Cox regression

Multivariable model included AJCC stage, age, R status, preoperative therapy, respiratory co-morbidity, cardiac co-morbidity and surgical approach.
